# Comparison of healthcare priorities in childhood and early/late adolescence: analysis of cross-sectional data from eight countries in the Council of Europe Child-friendly Healthcare Survey, 2011

**DOI:** 10.1111/cch.12169

**Published:** 2014-06-25

**Authors:** R Bensted, D S Hargreaves, J Lombard, U Kilkelly, R M Viner

**Affiliations:** ⋆ Royal Free London NHS Foundation TrustUK; † UCL Institute of Child HealthLondon, UK; ‡ University College CorkCork, Ireland

**Keywords:** adolescence, children's views, health services research

## Abstract

**Aims:**

To investigate healthcare priorities among children (≤12 years), early adolescents (13–15 years) and late adolescents (16–18 years).

**Methods:**

A total of 2023 respondents from eight European countries rated the importance of nine healthcare factors. The relative importance of these factors was compared within and between age groups, using mean score differences and logistic regression.

**Results:**

The most important item for all age groups was being listened to. Children rated pain control and the presence of parents more important than either understanding the doctor or being able to ask questions. Among adolescents, these differences disappeared for pain control and were reversed for parental presence. The changes in relative priorities between childhood and adolescence remained significant after adjusting for sex, long-standing illness and nationality (all *P* < 0.001).

**Conclusion:**

Healthcare priorities evolve significantly between childhood and early adolescence. However, being listened to is the most important priority at all ages.

## Background

In September 2011, the Council of Europe adopted Guidelines on Child-friendly Healthcare (Kilkelly 2011). These represent consensus around how healthcare services should incorporate the voice of children and young people and meet the specific needs of each age group. In England, the Children and Young People's Health Outcomes Forum (Lewis & Lenehan 2012) and the Kennedy report (Kennedy 2010) recommended that the voice of young patients should be promoted in the current National Health Service (NHS) reforms and that greater emphasis should be placed on providing age-appropriate care for adolescents.

Young adults are known to have distinct healthcare priorities from older adults (Hargreaves *et al*. 2012) and report a poorer experience of healthcare than other age groups (Kennedy 2010; Hargreaves & Viner 2012). However, little is known about how healthcare needs and priorities evolve during childhood and adolescence or about young people's views outside English-speaking countries (Kilkelly 2011).

To inform policy development, the Council of Europe commissioned a survey in 2011, inviting young people across its 47 member states to share their experiences and views about healthcare. Using these data, this study investigates how young people's priorities evolve with age.

## Methods

### Data

The questionnaire was translated into appropriate languages and administered by national partners of the Council of Europe during the Summer of 2011. The partner organizations disseminated the surveys through their links with healthcare services (including hospitals, primary care and dental care facilities), children's commissioner offices and non-governmental organizations working with and for children. In Finland, for example, the survey was administered by the Ombudsman for Children among 51 children either staying in hospital or attending outpatient services in Helsinki University Central Hospital, Hospital for Children and Adolescents and the children's ward at the Central Finland Central Hospital. The sampling strategy in other countries varied depending on the Council of Europe's national partners and their links with organizations connected to healthcare and children's services; some countries did not use a formal sampling frame and therefore no overall response rate is reported. The survey was also made available in 14 languages online and a small number of children completed the survey with this method. The main findings and details of the survey methodology have been published previously (Kilkelly 2011). The work of the Council of Europe is described more fully in Appendix I.

A total of 2257 valid questionnaires were returned from 22 countries. Fourteen countries accounted for very few responses (average of fewer than 20 completed questionnaires per country). These data were excluded because of increased risk of selection bias in such a small sample, and in order to permit adjustment for country in the logistic regression models. The final data set included 2023 young people from eight countries: Armenia, Austria, Bosnia and Herzegovina, England, Finland, Ireland, Malta, Spain. For analysis, respondents were grouped into children (<12 years), early adolescents (13–15 years) and later adolescents (16–18 years).

On a scale of 1 (not important at all) to 10 (very important), respondents were asked to rate the importance of nine questionnaire items. Item labels are included in brackets:

- having your parent/family with you *(parents)*;
- knowing the name of the doctor/nurse *(name)*;
- having treatment explained in advance/being prepared *(explanation)*;
- being able to understand what the doctor is saying *(understand)*;
- being able to ask questions *(questions)*;
- being listened to *(listened to)*;
- not being afraid *(unafraid)*;
- not being in pain *(pain)*;
- not feeling rushed *(unrushed)*.

### Analysis

Firstly, mean scores for each item were calculated, stratifying by age and sex. As all items were given higher scores by children than adolescents, we investigated the relative importance of items within each age group.

Preliminary analysis identified four items whose relative importance differed the most between age groups: *Parents* and *Pain* were rated more highly by younger respondents, while *Understanding* and *Questions* were rated more highly by adolescents.

Relative scores were compared for four pairs (*Parents vs. understanding*; *parents vs. questions*; *pain vs. understanding*; *pain vs. questions*), both within each age group and between age groups.

To minimize the risk of confounding, logistic regression was used to assess the statistical significance of comparisons between age groups, adjusting for sex, presence of a long-standing illness and nationality. A binary outcome was created, determined by whether the score for the first item in the pair (parents/pain) was greater than or equal to the second item (understanding/questions). We then calculated whether the odds ratio of the first item being rated more important than the second item differed between age groups, after adjusting for sex, the presence of a long-standing illness and nationality. To ensure that the results were not distorted by data from any one country, the regression analyses were repeated while omitting each country in turn.

### Ethics

No ethical approval was necessary as these are secondary analyses of previously published, anonymized data.

## Results

Characteristics of survey respondents are presented in Table 1.

**Table 1:** Characteristics of survey respondents from eight countries. Council of Europe survey, 2011

	*n*	%
Sex		
Male	1040	52.1
Female	954	47.9
Total	1996	100
Age		
Children under 10 years	151	7.5
Children 10–12 years	383	19
Early adolescents (13–15 years)	815	40.4
Late adolescents (16–18 years)	666	33.1
Total	2015	100
Long-standing illness		
No	1656	84.9
Yes	295	15.1
Total	1951	100
Country		
Armenia	201	9.9
Austria	1338	66.1
Bosnia	50	2.5
England	102	5
Finland	51	2.5
Ireland	178	8.8
Malta	30	1.5
Spain	73	3.6
Total	2023	100

**Figure 1:**
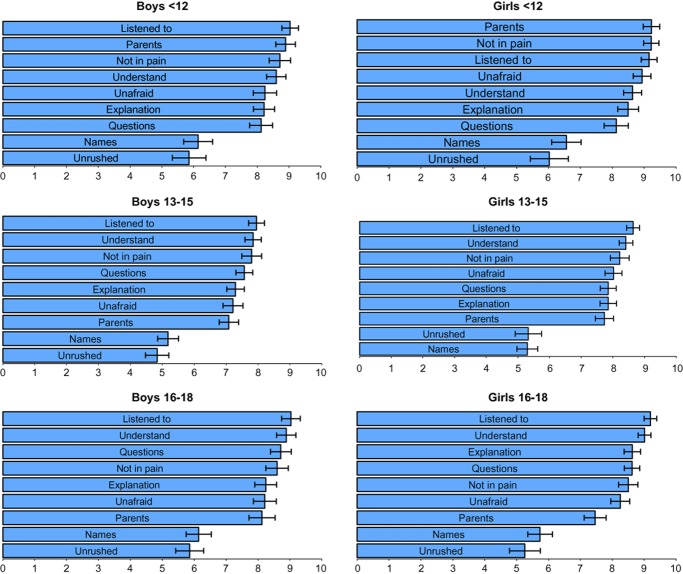
Importance ratings of nine healthcare factors by age and sex. Council of Europe survey, 2011. *Notes:* These bar charts show the mean importance score and 95% confidence intervals for each questionnaire item, by age and sex. Analyses are stratified into children (≤12 years), early adolescents (13–15 years) and late adolescents (16–18 years). See methods section for the full wording of questionnaire items.

Mean scores for each item by age and sex are presented in Fig. 1. On average, scores were higher for females than males; regarding age, they were highest among children, lowest for early adolescents and intermediate for later adolescents.

All age groups rated being listened to as the most important item. Knowing the names of professionals and not feeling rushed were consistently rated the least important items. As noted above, the relative importance of items relating to *Parents, Pain, Understanding* and *Questions* showed the greatest difference between age groups.

Children rated being with parents more important than understanding the doctor {9.0 vs. 8.6 [mean difference (MD) = 0.5 (95% confidence interval 0.2, 0.7)]}. This finding was reversed among early adolescents {7.4 vs. 8.1 [MD = −0.7 (−0.1.0, −0.5)]} and older adolescents {6.9 vs. 8.5 [MD = −1.7 (−2.0, −1.4)]}. Similarly, being with parents was rated more important than being able to ask questions for children {9.0 vs. 8.1 [MD = 0.9 (0.6, 1.2)] but no difference was found for early adolescents [7.4 vs. 7.7 (MD = −0.4 (−0.6, 0.1)]} and the reverse was found for older adolescents {6.9 vs. 8.3 [MD = −1.4 (−1.8, −1.1)]}.

Among children, pain control scored higher than understanding doctors {8.9 vs. 8.6 [MD = 0.3 (0.1, 0.5)]} and asking questions {8.9 vs. 8.1 [MD = 0.8 (0.5, 1.1)]}. There was no significant difference between these items for early adolescents {8.0 vs. 8.1 [MD = −0.1 (−0.3, 0.1)]} and {8.0 vs. 7.7 [MD = 0.2 (0.0, 0.5)]}, respectively. Among later adolescents, pain control was less important than understanding doctors {8.2 vs. 8.5 [MD = −0.4 (−0.6, −0.1)]} and equally important to asking questions {8.2 vs. 8.3 [MD = −0.2 (−0.5, 0.0)]}.

Scores for each item by age are presented in Appendix I (Table 2).

**Table 2:** Mean item scores by age and sex. Council of Europe survey, 2011

	Parents or family with you	Knowing professionals' name	Explanation preparation	Understanding the doctor	Able to ask questions	Being listened to	Not being afraid	Not feeling rushed	Not in pain
Children (≤12 years)									
Mean	9.04	6.32	8.33	8.60	8.12	9.07	8.53	5.94	8.93
SE Mean	0.10	0.16	0.12	0.11	0.13	0.09	0.12	0.20	0.11
Early adolescents (13–15 years)									
Mean	7.38	5.23	7.56	8.12	7.70	8.29	7.62	5.09	8.00
SE Mean	0.11	0.12	0.10	0.09	0.09	0.08	0.11	0.14	0.11
Late adolescents (16–18 years)									
Mean	6.87	5.47	8.08	8.50	8.29	8.75	7.80	4.92	8.16
SE Mean	0.14	0.14	0.11	0.10	0.11	0.09	0.12	0.17	0.12
Total									
Mean	7.65	5.59	7.93	8.37	8.00	8.64	7.92	5.25	8.29
SE Mean	0.07	0.08	0.06	0.05	0.06	0.05	0.07	0.09	0.07

The relative importance of the two items within each pair differed significantly between children and early adolescents (all *P* < 0.02). These differences remained significant after adjusting for sex, long-standing illness and nationality (all *P* < 0.001).

A significant difference between early and later adolescents was seen for the two comparisons relating to presence of parents (both *P* < 0.001 in the unadjusted analysis; *P* < 0.01 after adjustment). Differences were less significant between early and later adolescents when comparing pain control with asking questions and understanding doctors (*P* = 0.01 and 0.09 respectively unadjusted; both *P* > 0.1 after adjustment).

See Appendix I for full results of the regression analysis (Table 3).

**Table 3:** Odds ratios for selected healthcare priorities by age. Council of Europe survey, 2011

	Early adolescents vs. children		Late adolescents vs. children		Early vs. late adolescents	
AOR (95% CI)	*P*	AOR (95% CI)	*P*	AOR (95% CI)	*P*
Parents >= understanding doctors	0.24 (0.17, 0.35)	<0.001	0.17 (0.12, 0.25)	<0.001	1.41 (1.10, 1.82)	0.007
Parents >= asking questions	0.30 (0.21, 0.44)	<0.001	0.20 (0.13, 0.29)	<0.001	1.55 (1.19, 2.00)	0.001
Pain control >= asking questions	0.51 (0.35, 0.75)	<0.001	0.48 (0.33, 0.71)	<0.001	1.07 (0.80, 1.42)	0.644
Pain control >= understanding doctor	0.53 (0.37, 0.75)	<0.001	0.52 (0.36, 0.75)	<0.001	1.02 (0.77, 1.34)	0.914

Notes:

All odds ratios adjusted for sex, long-standing illness and nationality.

The symbol >= denotes that the importance rating for the first item was greater than or equal to the rating of the second item.

A value over 1 indicates greater odds of the first item scoring higher than the second. For example, early adolescents are more likely than late adolescents to value parental presence above understanding doctors.

Age groups were defined as children (≤12 years), early adolescents (13–15 years), late adolescents (16–18 years).

AOR, adjusted odds ratio; CI, confidence interval.

## Discussion

Our data show that feeling listened to was the most important item for all age groups. Although the importance of being listened to has previously been reported by many qualitative studies, we believe this is the first study to use quantitative data from such a diverse population. Young people using European health systems frequently feel that no-one listens to them (Kennedy 2010; Hargreaves & Viner 2012); it is hoped that a more robust evidence base about the importance of listening to young people will help to improve this situation in the future.

These data show that healthcare priorities evolve significantly between childhood and early adolescence, with young people aged 13–15 years reporting different priorities to younger children and more similar priorities to young adults. Again, we believe this study is the first to use multinational, quantitative data to confirm similar findings from small, qualitative studies (Kennedy 2010).

Early adolescents aged 13–15 are frequently treated in the same way as younger children, with little recognition of their growing desire for autonomy. For example, during early adolescence, young people with long-term medical conditions in England frequently receive little support to learn self-care skills and the confidence to manage relationships with professionals, contributing to avoidable anxiety and poor outcomes in early adulthood.

### Strengths and limitations

Strengths of this study include the large, diverse sample. Unlike many previous published studies, the participants were not restricted to hospital patients, young people with a specific condition or those from English-speaking countries. Although unequal numbers of young people were recruited from different countries, no difference in results was seen when excluding participants from any single country.

The findings are recent and directly relevant to health policy in England and other European countries. Lastly, the questionnaire design allows direct comparison of the importance of difference aspects of care, rather than indirect measures used in some previous studies of healthcare priorities (Hargreaves *et al*. 2012).

The principal weakness is the differing sample size and sampling strategy in each country. The survey was intended to reflect the views of all children and young people living in Council of Europe countries; the sampling strategy therefore focused on inclusivity rather than ensuring a nationally representative sample from each country. As a result, it is unfortunately not possible to make cross-country comparisons or to report an overall response rate.

There may well be a degree of selection bias among those who responded; however, this weakness is mitigated by the consistency of findings across eight different countries with very different healthcare services and cultural contexts. We note that formal national surveys in England have reported very low response rates among young people, suggesting that use of a formal sampling frame does not exclude the risk of substantial selection bias, especially in this age group.

At country level, the number of completed surveys appeared to reflect the resources and capacity of the Council of Europe partner organizations, rather than geographical or cultural differences. In view of the consistency of our findings across such diverse populations, we therefore believe that these findings are broadly generalizable across the Council of Europe countries, and perhaps more widely.

A further weakness is that some healthcare issues identified in other studies as important to young people could not be included. These include privacy and confidentiality (Hargreaves *et al*. 2012).

## Conclusions

Feeling listened to was rated the most important feature of health services throughout childhood and adolescence. The relative importance of other factors changed significantly between childhood and early adolescence. Children rated the presence of parents/family more highly than understanding the doctor or being able to ask questions, while the reverse was seen for adolescents. The relative importance of pain control was also higher among children than adolescents.

## Key messages

- Adolescence is now recognized as a key life-stage where lifelong attitudes to healthcare and health-related behaviours are acquired.
- Young people report the lowest satisfaction rates with healthcare of any age group, but their specific needs and priorities are not well understood.
- This multi-national, quantitative study found that feeling listened to was rated the most important priority for all participants, from 8 to 18 years.
- Early adolescents had distinct priorities from younger children, with much more importance given to good communication and being able to ask questions.
- Services which listen to young people and recognize the distinct needs of early adolescents may improve outcomes among this sometimes neglected group.

## Appendix I

### Background to the Council of Europe survey on child-friendly healthcare

The Council of Europe is an international organization committed to the protection of human rights and the rule of law in Europe. It plays a leading role in the development and agreement of international standards in a range of areas including healthcare and children's rights. Notwithstanding the many logistical and methodological challenges involved, the Council has recently sought to incorporate the views of children and young people directly into its law-making activities. During the drafting of the Guidelines on Child-friendly Healthcare, qualitative research identified knowledge gaps in what is known about children's views about healthcare and it was decided to survey children and young people across the Council of Europe on these issues. The questionnaire was developed at University College Cork and piloted among a small group of children in Ireland. The survey was then circulated widely among the Council of Europe's partners at national level – including governmental and non-governmental organizations concerned with children and health – with ethical and practical guidance as to how the survey was to be administered. The survey was translated into several languages and placed on the Council of Europe website although only a handful of completed surveys were completed online. Further details of the survey methodology and results are available from the Council of Europe website (Kilkelly 2011).
